# Longitudinal atrophy in early Braak regions in preclinical Alzheimer's disease

**DOI:** 10.1002/hbm.25151

**Published:** 2020-08-26

**Authors:** Long Xie, Laura E. M. Wisse, Sandhitsu R. Das, Nicolas Vergnet, Mengjin Dong, Ranjit Ittyerah, Robin de Flores, Paul A. Yushkevich, David A. Wolk, for the Alzheimer's Disease Neuroimaging Initiative

**Affiliations:** ^1^ Penn Image Computing and Science Laboratory (PICSL) University of Pennsylvania Philadelphia Pennsylvania USA; ^2^ Department of Radiology University of Pennsylvania Philadelphia Pennsylvania USA; ^3^ Department of Diagnostic Radiology Lund University Lund Sweden; ^4^ Department of Neurology University of Pennsylvania Philadelphia Pennsylvania USA; ^5^ Penn Memory Center University of Pennsylvania Philadelphia Pennsylvania USA

**Keywords:** ASHS, Brodmann area 35, cross‐sectional, entorhinal cortex, hippocampus, longitudinal atrophy, MRI, preclinical Alzheimer's disease, tau

## Abstract

A major focus of Alzheimer's disease (AD) research has been finding sensitive outcome measures to disease progression in preclinical AD, as intervention studies begin to target this population. We hypothesize that tailored measures of longitudinal change of the medial temporal lobe (MTL) subregions (the sites of earliest cortical tangle pathology) are more sensitive to disease progression in preclinical AD compared to standard cognitive and plasma NfL measures. Longitudinal T1‐weighted MRI of 337 participants were included, divided into amyloid‐β negative (Aβ−) controls, cerebral spinal fluid p‐tau positive (T+) and negative (T−) preclinical AD (Aβ+ controls), and early prodromal AD. Anterior/posterior hippocampus, entorhinal cortex, Brodmann areas (BA) 35 and 36, and parahippocampal cortex were segmented in baseline MRI using a novel pipeline. Unbiased change rates of subregions were estimated using MRI scans within a 2‐year‐follow‐up period. Experimental results showed that longitudinal atrophy rates of all MTL subregions were significantly higher for T+ preclinical AD and early prodromal AD than controls, but not for T− preclinical AD. Posterior hippocampus and BA35 demonstrated the largest group differences among hippocampus and MTL cortex respectively. None of the cross‐sectional MTL measures, longitudinal cognitive measures (PACC, ADAS‐Cog) and cross‐sectional or longitudinal plasma NfL reached significance in preclinical AD. In conclusion, longitudinal atrophy measurements reflect active neurodegeneration and thus are more directly linked to active disease progression than cross‐sectional measurements. Moreover, accelerated atrophy in preclinical AD seems to occur only in the presence of concomitant tau pathology. The proposed longitudinal measurements may serve as efficient outcome measures in clinical trials.

## Graphical abstract

We investigated the utility of tailored measures of longitudinal change of the medial temporal lobe (MTL) subregions quantified from longitudinal structural MRI in tracking disease progression in preclinical Alzheimer's disease. Experiment results showed that our longitudinal measurements are more sensitive to disease progression in preclinical AD compared to longitudinal cognitive (PACC, ADAS‐Cog) and plasma NfL measures. Also, accelerated atrophy in preclinical AD seems to occur only in the presence of concomitant tau pathology. The proposed longitudinal measurements may serve as efficient outcome measures in clinical trials.

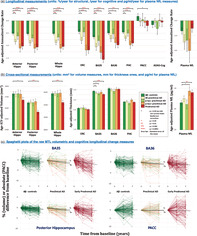

## 1 INTRODUCTION

As intervention studies in Alzheimer's disease (AD) have moved towards the preclinical stage (Sperling et al., 2011), a major challenge has been finding sensitive outcome measures for intervention studies. Structural MRI is a promising candidate because, compared to PET, it is more accessible, less expensive and has higher resolution and lower repeat measurement error. Longitudinal change in the medial temporal lobe (MTL), the earliest region affected by neurofibrillary tangle (NFT) pathology, quantified from structural MRI has repeatedly demonstrated sensitivity for early diagnosis and monitoring of patients with mild cognitive impairment (MCI; Chincarini et al., 2016; Iglesias et al., 2016; Jack et al., 2000; Kulason et al., 2019; Ledig, Schuh, Guerrero, Heckemann, & Rueckert, 2018; Leung et al., 2010; Morra et al., 2009; Schuff et al., 2009; Tward et al., 2017; Wolz et al., 2010). However, there is limited evidence that the more subtle longitudinal change in preclinical AD can be reliably detected with longitudinal structural MRI.

Four recent prospective papers have evaluated the sensitivity of longitudinal MRI to preclinical AD. Donohue et al. (2017) reported a significant difference in hippocampal volume change between amyloid‐β positive (Aβ+) and negative (Aβ−) cognitively normal individuals. However, this effect reached significance only after 4 years and was weaker than that of the preclinical Alzheimer's cognitive composite (PACC) score, a standard cognitive measure used in preclinical AD. Conversely, Pegueroles et al. (2017) reported significant atrophy rate difference in the MTL using only scans within 2‐year follow‐up in a relatively small sample of preclinical AD patients compared to controls. Similarly in a small sample of preclinical AD with evidence of tau pathology, Holland, McEvoy, Desikan, and Dale (2012) found significant differences in longitudinal atrophy rates compared to Aβ− controls over a 3‐year period. However, both these two studies did not report how their measurements compared with cognitive scores and replication in larger cohorts needs to be done. A significant interaction between cerebrospinal fluid (CSF) Aβ, phospho‐tau biomarkers and cross‐sectional cortical thickness in preclinical AD was observed by Fortea et al. (2014), but they did not report a significant thickness difference between Aβ+ and Aβ− groups. Additionally, several retrospective studies have reported longitudinal and cross‐sectional structural changes in cognitively normal individuals who progressed to cognitive impairment (Miller et al., 2013; Roe et al., 2018; Younes et al., 2019). In particular, Miller et al. (2013) found significantly increased atrophy rates in the hippocampus and entorhinal cortex of cognitively normal individuals who progressed to MCI. This suggests that MTL longitudinal markers are sensitive to asymptomatic disease, but since clinical trials do not have access to information on who will or will not progress to cognitive impairment, it remains critical to evaluate the sensitivity of longitudinal MRI to preclinical AD, as defined by Aβ positivity, in a prospective setting.

**FIGURE 1:**
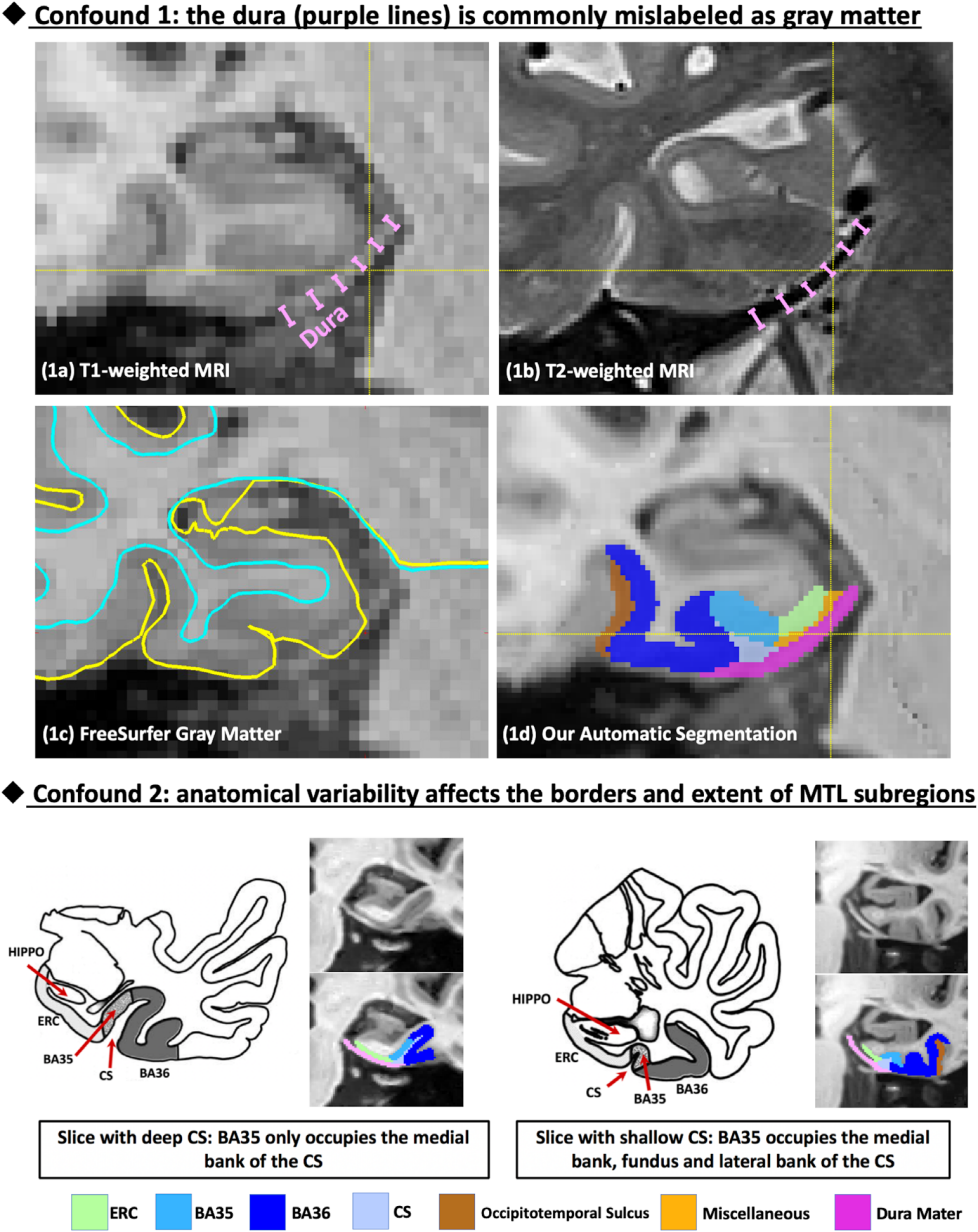
Common confounds in automatic segmentation of medial temporal lobe (MTL) subregions using T1‐weighted MRI. Confound 1: the dura mater (indicated by purple lines) has similar intensity with gray matter (GM) in T1‐weighted MRI (a) but can be easily separated in T2‐weighted MRI (b), is commonly mislabeled as GM (c). Confound 2: Large anatomical variability exists in the MTL defined by the pattern of the collateral sulcus (CS), which influences the borders and extent of the subregions of the MTL cortex. Our segmentation pipeline is able to reliably separate dura from GM (1d) and account for anatomical variability. Figure adapted from Ding and Van Hoesen (2010). BA, Brodmann area; ERC, entorhinal cortex; HIPPO, hippocampus

Several barriers that may hinder the sensitivity of structural measures in preclinical AD: (a) Most studies have been cross‐sectional, which may be suboptimal because they are influenced by nondisease effects, such as inter‐subject variability due to developmental and other lifespan factors. Longitudinal measurement reflects active neurodegeneration and thus should be more sensitive to evidence of underlying AD pathology. (b) Most studies have not included Brodmann area 35, which approximates the transentorhinal cortex, the earliest area of NFT pathology (Braak & Braak, 1995). (c) Measurements of the MTL cortical subregions have associated confounds (cortex over‐segmentation due to the dura and error in subregion boundaries due to anatomical variability; Figure 1) limiting measurement accuracy. In this study, we hypothesize that a tailored MRI computational pipeline focused on MTL subregions will yield longitudinal biomarkers, measured within a practical timeframe for theoretical clinical trial (2‐year follow‐up), that are more sensitive to disease progression in preclinical AD than standard cognitive measures. We also wanted to compare to an emerging blood based biomarker, plasma neurofilament light chain (NfL), which has displayed considerable promise as a potentially easily accessible biomarker of neurodegeneration (Mattsson et al., 2017).

## 2 METHODS

This section provides information on participants, data processing pipeline and statistical analysis. Details on the Alzheimer's Disease Neuroimaging Initiative (ADNI) study, MRI acquisition, quality control, statistical analyses and sample size estimation are available in Supplementary S1.

### 2.1 Participants

Participants from the ADNI‐GO and ADNI‐2 studies who had longitudinal T1‐weighted (T1w) MRI and Florbetapir PET scans at baseline available were included. To simulate a realistic clinical trial, only longitudinal scans within 2‐year follow‐up of baseline were analyzed for each participant. Participants who had no scans beyond 1 year after baseline were excluded, since longitudinal atrophy over only 1 year is unlikely to be detected in preclinical AD. A summary standardized uptake value ratio (SUVR) derived from Florbetapir PET^1^ was used to determine the Aβ status of each participant (threshold of 1.11; Landau et al., 2012). After quality control (Supplementary S1.3), 337 participants (summarized in Table 1) were selected and grouped into Aβ− cognitively normal controls, preclinical AD (Aβ+ controls), early prodromal AD (Aβ+ early MCI, or EMCI). To investigate whether the presence of tau pathology is associated with rate of neurodegeneration in preclinical AD, we further divided this group into tau positive (T+) and negative (T−) subgroups based on CSF p‐tau measurement (threshold of 23 pg/mL; Shaw et al., 2009, 68 out of 76 preclinical AD patients have CSF p‐tau measurements available).

**TABLE 1:** Dataset characteristics

	Aβ− control	Preclinical AD (Aβ+ control)			Early prodromal AD (Aβ+ EMCI)
		CSF p‐tau negative (T−)	CSF p‐tau positive (T+)	Whole group	
Number of subjects	151	32	36	76	110
Age (years)	71.5 (6.2)	74.3 (5.9)*	74.7 (5.5)**	74.4 (5.8)***	72.8 (6.9)
Sex (male/female)	83/68	12/20	10/26**	25/51**	63/47
Education (years)	16.9 (2.3)	16.5 (2.6)	15.9 (2.9)*	16.1 (2.8)*	15.7 (2.8)***
MMSE	29.1 (1.2)	28.9 (1.0)	29.2 (0.9)	29.1 (0.9)	28.0 (1.7)***
Mean number of timepoints	4.3 (1.0)	4.1 (1.2)	3.9 (1.3)	4.0 (1.2)	4.8 (0.5)**

*Notes*: All statistics are in comparison to Aβ− control. *SD* is reported in parenthesis. Independent two‐sample *t*‐test (continuous variables with normal distribution, including age, education, and mean number of timepoints), Mann–Whitney *U* test (continuous variable with nonnormal distribution, i.e., MMSE) and contingency *χ*^2^ test (sex) were performed.

Abbreviations: AD, Alzheimer's disease; Aβ, amyloid‐β; CSF, cerebrospinal fluid; EMCI, early mild cognitive impairment; MMSE, mini‐mental state examination.

*p < 0.05; **p < 0.01; ***p < 0.001.

### 2.2 Cross‐sectional and longitudinal quantitative measures of MTL subregions

The longitudinal MRI scans were processed using a tailored pipeline (summarized in Figure S1) that accounts for common confounds of conventional approaches (described in Figure 1). A multi‐atlas automatic segmentation algorithm “ASHS‐T1”^2^ (Xie et al., 2016; Xie et al., 2019) was used to label the anterior and posterior hippocampus, entorhinal cortex (ERC), Brodmann areas (BA) 35 and 36, and parahippocampal cortex (PHC) in each baseline MRI scan.

For cross‐sectional analysis, the volume of the anterior and posterior hippocampus and the median thickness of MTL cortical subregions (ERC, BA35, BA36, and PHC) were extracted from the segmentations (Xie et al., 2017; Xie et al., 2018). For longitudinal analysis, symmetric diffeomorphic registration (Avants, Epstein, Grossman, & Gee, 2008) was performed between the baseline MRI scan and each of the follow‐up MRI scans. The volume of each MTL subregion in each follow‐up scan was estimated by applying the spatial transformation computed by the registration to the ASHS‐T1 segmentation of the baseline scan in a manner that is unbiased (Das et al., 2012). For each subregion in each subject, the annualized volume atrophy rate was computed by linear regression of all available longitudinal volume measurements vs. scan date differences from baseline.

This was then converted to a relative volume atrophy rate (in %) by dividing by the baseline subregion volume. Bilateral measurements of each subregion were averaged to increase reliability. The conclusions do not change when using measurements of left or right hemisphere separately.

### 2.3 Cognitive and plasma NfL data processing

To compare our longitudinal measurements with cognitive measurements commonly used in preclinical and prodromal AD and an alternative blood‐based neurodegeneration biomarker, we included the PACC (computed as in Donohue et al., 2017) using standardized z score composite of the ADAS‐Cog subscale delayed word recall, delayed recall score on logical memory test, MMSE, and the log‐transformed trail‐making test B time to completion), Alzheimer's Disease Assessment Scale‐Cognitive (ADAS‐Cog, ADAS11‐Cog was used) and plasma NfL. All measurements are publicly available from the ADNI database. Annualized longitudinal rate of change was computed using linear regression in the same manner as in Section 2.2. Since we found that normalizing by baseline measures did not improve discriminability, we used the absolute change rate of these measurements.

### 2.4 Statistical analysis

The longitudinal and cross‐sectional measurements (neuroimaging, cognitive and plasma NfL) of each patient group were compared to Aβ− controls separately using general linear models with each measurement as the dependent variable, group membership as the factor of interest, and age as covariate. Intracranial volume was included as an additional covariate for cross‐sectional volume measurements. Sex was not included as a covariate in this study, but including it did not significantly change the reported findings. Holm–Bonferroni correction for multiple comparisons was performed (Holm, 1979). In addition, we estimated the sample size (details in Supplementary S1.4) required to detect both 50%/year and 25%/year reduction in the atrophy rate of each patient group relative to that of Aβ− controls (power 1 − *β* = 0.8, one‐sided significance level *α* = 0.05). The 95% confidence interval of each sample size estimate was computed using the bootstrap method (Efron, 1979). Since we have a strong hypothesis on the direction of the effect, the above analyses were one‐sided.

## 3 RESULTS

**FIGURE 2:**
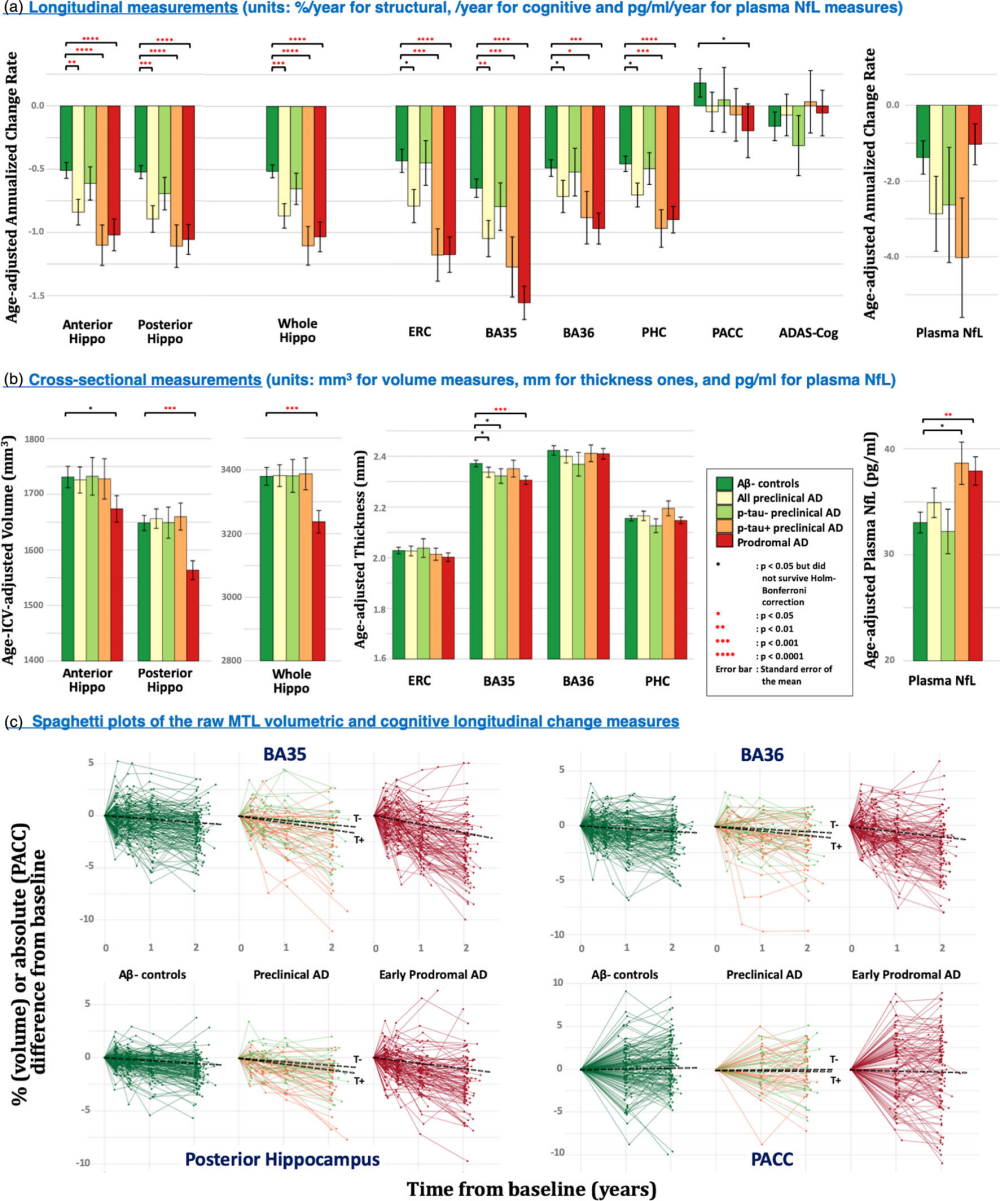
Comparisons of longitudinal (a) and cross‐sectional (b) measurements in discriminating patient groups from Aβ− controls. Spaghetti plots of the raw longitudinal change measurements relative to baseline of representative medial temporal lobe (MTL) subregions and PACC are shown in (c) and the black dashed lines indicate the fitted mean longitudinal change. All the statistical tests are one‐sided. Coloring scheme for the five groups are consistent in the three subplots. AD, Alzheimer's disease; ADAS‐Cog, Alzheimer's Disease Assessment Scale‐Cognitive; Aβ, amyloid‐β; BA35/36, Brodmann area 35/36; ERC, entorhinal cortex; Hippo, hippocampus; NfL, neurofilament light chain; PACC, preclinical Alzheimer's cognitive composite score; PHC, parahippocampal cortex

The results of group comparisons using longitudinal and cross‐sectional measurements are summarized in Figure 2 and described in detail below.

### 3.1 Longitudinal change in MTL subregion volume and cognition

As shown in Table 2 and Figure 2a, we observed Holm–Bonferroni‐corrected significant group differences between the whole preclinical AD group and Aβ− controls in anterior hippocampus (*F* = 7.9, *p* = 2.7 × 10^−3^), posterior hippocampus (*F* = 12.5, *p* = 2.6 × 10^−4^), whole hippocampus (*F* = 12.1, *p* = 3.1 × 10^−4^) and BA35 (*F* = 7.7, *p* = 2.9 × 10^−3^). When further dichotomizing preclinical AD based on CSF p‐tau status, significant groups differences were observed in the atrophy rates of all MTL subregions except BA36 between T+ preclinical AD and Aβ− controls with posterior hippocampus (*F* = 18.7, *p* = 1.8 × 10^−5^) and ERC (*F* = 12.2, *p* = 3.0 × 10^−4^) displaying the largest effect size in hippocampus and MTL cortex respectively. This pattern was also present in early prodromal AD, but with a larger effect size than in the T+ preclinical group and with BA35 being the strongest MTL region (*F* = 41.2, *p* = 3.4 × 10^−10^). No significant difference was observed in T− preclinical AD. In contrast, neither of the two cognitive measures, as well as the plasma NfL measure, produced Holm‐Bonferroni‐corrected significant group differences in any disease group, although we did observe a trend using PACC (*F* = 2.9, *p* = .045 uncorrected) in the early prodromal AD group.

**TABLE 2:** Statistical analysis results using longitudinal atrophy measures of the medial temporal lobe subregions, longitudinal cognitive and plasma NfL measures, adjusted for age, in discriminating patients from Aβ− control

Measurement	Aβ− control (A− controls)	Preclinical AD			Early prodromal AD (A+ EMCI)
		CSF p‐tau negative (A + T− controls)	CSF p‐tau positive (A + T+ controls)	Whole group (A+ controls)	
Total *n*	151	32	36	76	110
Annualized volume change rate (%/year), *SD* in parentheses					
Anterior hippo	−0.51 (0.75)	−0.61 (0.74)	***−1.10 (0.94)***	***−0.84 (0.88)***	***−1.02 (1.27)***
*n*	144	32	***35***	***74***	***102***
*F* stats		<2.5	***14.7***	***7.9***	***15.7***
*p* value		*>.1*	***8.9e−5***	***2.7e−3***	***4.9e−5***
Cohen's *d*		0.14	**0.69**	**0.40**	**0.49**
Posterior hippo	−0.52 (0.62)	−0.69 (0.73)	***−1.11 (1.00)***	***−0.89 (0.91)***	***−1.06 (1.19)***
*n*	144	32	***35***	***74***	***102***
*F* stats		<2.5	***18.7***	***12.5***	***20.4***
*p* value		*>.1*	***1.8e−5***	***2.6e−4***	***5.0e−6***
Cohen's *d*		0.25	**0.71**	**0.48**	**0.56**
Whole hippo	−0.51 (0.62)	−0.65 (0.71)	***−1.11 (0.90)***	***−0.87 (0.84)***	***−1.03 (1.19)***
*n*	144	32	***35***	***74***	***102***
*F* stats		<2.5	***19.9***	***12.1***	***19.8***
*p* value		*>.1*	***7.0e−6***	***3.1e−4***	***7.0e−6***
Cohen's *d*		0.21	**0.76**	**0.48**	**0.55**
ERC	−0.43 (1.12)	−0.45 (1.00)	***−1.18 (1.25)***	−0.79 (1.15)	***−1.18 (1.47)***
*n*	150	32	***36***	*76*	***110***
*F* stats		<2.5	***12.2***	*5.0*	***21.7***
*p* value		*>.1*	***3.0e−4***	*.013*	***2.5e−6***
Cohen's *d*		0.02	**0.63**	0.32	**0.57**
BA35	−0.65 (0.89)	−0.80 (1.07)	***−1.27 (1.43)***	***−1.05 (1.25)***	***−1.56 (1.38)***
*n*	150	32	***36***	***76***	***110***
*F* stats		<2.5	***11.1***	***7.7***	***41.2***
*p* value		*>.1*	***5.3e−4***	***2.9e−3***	***3.4e−10***
Cohen's *d*		0.15	**0.52**	**0.37**	**0.78**
BA36	−0.49 (0.81)	−0.47 (1.07)	−0.83 (1.27)	−0.66 (1.13)	***−0.95 (1.30)***
*n*	150	32	36	76	***110***
*F* stats		<2.5	5.2	2.8	***13.5***
*p* value		*>.1*	*.012*	*.049*	***1.5e−4***
Cohen's *d*		0.02	0.32	0.18	**0.42**
PHC	−0.45 (0.75)	−0.44 (0.73)	**−0.91 (0.90)**	−0.65 (0.84)	***−0.87 (1.11)***
*n*	150	32	***36***	76	***110***
*F* stats		<2.5	***11.8***	5.1	***14.7***
*p* value		*>.1*	***3.6e−4***	*.013*	***7.9e−5***
Cohen's *d*		0.01	**0.56**	0.25	**0.45**
Annualized change rates of other markers of neurodegeneration (/year), *SD* in parentheses					
Plasma NfL	−1.38 (5.23)	−2.63 (8.20)	−4.02 (9.05)	−2.86 (8.30)	−1.03 (5.38)
*n*	141	29	33	70	99
*F* stats		<2.5	<2.5	<2.5	<2.5
*p* value		*>.1*	*>.1*	*>.1*	*>.1*
Cohen's *d*		0.18	0.36	0.21	0.07
Annualized change rates of cognitive measurements (/year), *SD* in parentheses					
PACC	0.18 (1.38)	0.05 (1.44)	−0.07 (1.24)	−0.05 (1.34)	−0.20 (2.21)
*n*	151	32	36	76	108
*F* stats		<2.5	<2.5	<2.5	2.9
*p* value		*>.1*	*>.1*	*>.1*	*.045*
Cohen's *d*		0.09	0.19	0.17	0.21
ADAS‐Cog	−0.16 (1.38)	−0.31 (1.35)	0.03 (1.47)	−0.07 (1.43)	−0.06 (1.87)
*n*	150	32	36	76	108
*F* stats		<2.5	<2.5	<2.5	<2.5
*p* value		*>.1*	*>.1*	*>.1*	*>.1*
Cohen's *d*		0.11	0.14	0.06	0.06

*Note:* The preclinical AD was further dichotomized based on CSF p‐tau (threshold: 23 pg/mL). Bilateral measurements of each subregion were averaged. Negative change indicates the measurement change towards worse condition in follow‐ups. Measurements that survived Holm‐Bonferroni correction are highlighted in bold font. Number of measurements of anterior/posterior hippocampus and MTL cortical subregions are presented based on exclusions on some measures due to quality control (Supplementary S1.3). All the statistical tests are one‐tailed.

Abbreviations: AD, Alzheimer's disease; ADAS‐Cog, Alzheimer's Disease Assessment Scale‐Cognitive; BA35/36, Brodmann area 35/36; CSF, cerebrospinal fluid; EMCI, early mild cognitive impairment; ERC, entorhinal cortex; Hippo, hippocampus; NfL, neurofilament light chain; PACC, preclinical Alzheimer's cognitive composition score; PHC, parahippocampal cortex.

In order to visualize the raw data, spaghetti plots of representative longitudinal change measurements relative to baseline, including posterior hippocampus (largest effect size in the hippocampus), BA35 volume (largest effect size in MTL cortex), BA36 (subregion in the MTL least affected in early stages of AD) and PACC are shown in Figure 2c.

### 3.2 Cross‐sectional volume/thickness and plasma NfL differences between patients and Aβ− controls

The group comparison results using cross‐sectional measurements of MTL subregions and plasma NfL measurements are summarized in Table 3 and Figure 2b. Compared to longitudinal MTL subregional measurements, cross‐sectional discrimination is much weaker, with no Holm‐Bonferroni‐corrected significant differences observed in preclinical AD (including the T+ preclinical AD subgroup). Only BA35 displayed a *p*‐value <.05 before correction in the whole preclinical AD sample compared to controls (*F* = 3.5, *p* = .035), which appears to be driven by the T− preclinical AD individuals (*F* = 3.9, *p* = .025) rather than the T+ ones (although both groups had more cortical thinning than controls in absolute terms). In the early prodromal stage, significant reduction in posterior (*F* = 15.4, *p* = 5.7 × 10^−5^) and whole hippocampus volume (*F* = 10.1, *p* = 8.3 × 10^−4^) and BA35 thickness (*F* = 10.6, *p* = 6.5 × 10^−4^) were found, but with smaller effect size than that of the corresponding longitudinal measures. On the other hand, cross‐sectional plasma NfL measurements perform better, compared to longitudinal NfL ones, in separating early prodromal AD (*F* = 8.7, *p* = 1.8 × 10^−3^) and T+ preclinical AD patients (*F* = 5.3, *p* = .011, trend level significance) from Aβ− controls.

**TABLE 3:** Statistical analysis results using cross‐sectional measures of the medial temporal lobe subregions, adjusted for age (all measurements) and intracranial volume (volume measurements), in discriminating patient from Aβ− control

Measurement	Aβ− control	Preclinical AD			Early prodromal AD (A+ EMCI)
		CSF p‐tau negative (A + T− controls)	CSF p‐tau positive (A + T+ controls)	Whole group (A+ controls)	
Total *n*	151	32	36	76	110
Volume (mm^3^), adjusted for age and intracranial volume, *SD* in parentheses					
Anterior hippo	1731.12 (230.70)	1732.26 (190.60)	1727.70 (214.80)	1725.77 (202.00)	1,673.87 (238.87)
% difference		0.1	−0.2	−0.3	−3.3
*n*	144	32	*35*	*74*	*102*
*F* stats		*<2.5*	*<2.5*	*<2.5*	*3.5*
*p* value		*>.1*	*>.1*	*>.1*	*.032*
Cohen's *d*		0.01	0.02	0.02	0.24
Posterior hippo	1,648.64 (164.33)	1,649.04 (157.50)	1,659.97 (143.36)	1,656.33 (150.76)	**1,563.61 (172.13)**
% difference		0.0	0.7	0.5	**−5.2**
*n*	144	32	*35*	*74*	***102***
*F* stats		*<2.5*	*<2.5*	*<2.5*	***15.4***
*p* value		*>.1*	*>.1*	*>.1*	***5.7e−5***
Cohen's *d*		0.00	0.07	0.05	**0.51**
Whole hippo	3,379.77 (333.03)	3,381.27 (292.12)	3,387.64 (295.23)	3,382.06 (292.39)	**3,237.50 (357.68)**
% difference		0.0	0.2	0.1	**−4.2**
*n*	144	32	*35*	*74*	***102***
*F* stats		*<2.5*	*<2.5*	*<2.5*	***10.1***
*p* value		*>.1*	*>.1*	*>.1*	***8.3e−4***
Cohen's *d*		0.00	0.03	0.01	**0.41**
Thickness (mm), adjusted for age, *SD* in parentheses					
ERC	2.03 (0.16)	2.04 (0.21)	2.01 (0.14)	2.03 (0.17)	2.00 (0.17)
% difference		0.4	−0.7	−0.1	−1.3
*n*	150	32	*36*	*76*	*110*
*F* stats		*<2.5*	*<2.5*	*<2.5*	*<2.5*
*p* value		*>.1*	*>.1*	*>.1*	*>.1*
Cohen's *d*		0.05	0.10	0.01	0.16
BA35	2.37 (0.16)	2.32 (0.16)	2.35 (0.20)	2.34 (0.17)	**2.31 (0.17)**
% difference		−2.1	−0.9	−1.5	**−2.8**
*n*	150	32	*36*	*76*	***110***
*F* stats		3.9	*<2.5*	*3.5*	***10.6***
*p* value		*.025*	*>.1*	*.035*	***6.5e−4***
Cohen's *d*		0.31	0.12	0.21	**0.41**
BA36	2.42 (0.23)	2.37 (0.26)	2.41 (0.20)	2.40 (0.22)	2.41 (0.22)
% difference		−2.3	−0.5	−1.0	−0.6
*n*	150	32	36	76	*110*
*F* stats		*<2.5*	*<2.5*	*<2.5*	*<2.5*
*p* value		*>.1*	*>.1*	*>.1*	*>.1*
Cohen's *d*		0.22	0.05	0.11	0.06
PHC	2.16 (0.12)	2.13 (0.15)	2.19 (0.18)	2.17 (0.16)	2.15 (0.14)
% difference		−1.3	1.8	0.5	−0.4
*n*	150	32	*36*	76	*110*
*F* stats		*<2.5*	*<2.5*	*<2.5*	*<2.5*
*p* value		*>.1*	*>.1*	*>.1*	*>.1*
Cohen's *d*		0.21	0.26	0.07	0.07
Other markers of neurodegeneration, adjusted for age, *SD* in parentheses					
Plasma NfL (pg/mL)	−33.06 (11.91)	−32.21 (11.70)	−38.65 (11.95)	−34.92 (12.02)	**−37.91 (13.68)**
% difference		−2.6	16.9	5.6	**14.7**
*n*	147	31	*36*	*75*	***106***
*F* stats		*<2.5*	*5.3*	*<2.5*	***8.7***
*p* value		*>.1*	*.011*	*>.1*	***1.8e−3***
Cohen's *d*		0.07	0.47	0.16	0.38

*Note:* The preclinical AD was further dichotomized based on CSF p‐tau (threshold: 23 pg/mL). Bilateral measurements of each subregion were averaged. Measurements that survived Holm–Bonferroni correction are highlighted in bold font. Number of measurements of anterior/posterior hippocampus and MTL cortical subregions is because we excluded some of the measurements in quality control (Supplementary S1.3) All the statistical tests are one‐tailed.

Abbreviations: AD, Alzheimer's disease; BA35/36, Brodmann area 35/36; CSF, cerebrospinal fluid; EMCI, early mild cognitive impairment; ERC, entorhinal cortex; Hippo, hippocampus; NfL, neurofilament light chain; PHC, parahippocampal cortex.

### 3.3 Sample size estimations

The sample size required to detect both 50%/year and 25%/year reduction in atrophy rate is reported in Table 4. For preclinical AD, the whole hippocampus yields the smallest sample size estimate in absolute terms, both when considering the whole preclinical AD group (278/1113 for 50/25%/year) and the T+ subgroup (115/460 for 50/25%/year). For early prodromal AD, BA35 yields the smallest estimate (114/455 for 50/25%/year). Cognitive and plasma NfL measurements would require at least five times more subjects compared to the corresponding best longitudinal MRI measures.

**TABLE 4:** Sample size (95% confidence interval in parenthesis) required to detect both 50%/year and 25%/year reduction in change rate of each patient group compared to that of Aβ− controls (power 1 − *β* = 0.8, one‐sided significance level *α* = 0.05)

% change	Measurements	Preclinical AD		Early prodromal AD (A+ EMCI)
		CSF p‐tau positive (A + T+ controls)	Whole group (A+ controls)	
50%	Longitudinal atrophy rate			
	Anterior hippo	125 (52, 454)	345 (126, 2,784)	305 (118, 1,504)
	Posterior hippo	142 (57, 593)	295 (112, 1795)	246 (95, 939)
	Whole hippo	**115 (48, 394)**	**278 (109, 1,518)**	259 (106, 1,050)
	ERC	137 (49, 736)	506 (145, 1.8 × 10^4^)	192 (84, 621)
	BA35	258 (84, 2,567)	482 (157, 7,025)	**114 (56, 274)**
	BA36	701 (157, 1.4 × 10^5^)	2087 (335, 7.2 × 10^5^)	395 (141, 2,723)
	PHC	190 (62, 1,520)	871 (200, 1.1 × 10^5^)	342 (116, 2046)
	Longitudinal change in other markers of neurodegeneration			
	Plasma NfL	575 (134, 4.6 ×10^4^)	1,534 (348, 2.8 ×10^5^)	11,712 (401, 2.2 ×10^6^)
	Longitudinal change in cognition			
	PACC	1,197 (155, 4.0×10^5^)	1709 (257, 5.7×10^5^)	1,675 (335, 3.1×10^5^)
	ADAS‐Cog	2,865 (183, 8.8×10^5^)	12,646 (409, 2.4 × 10^6^)	15,713 (617, 2.6 × 10^6^)
25%	Longitudinal atrophy rate			
	Anterior hippo	501 (211, 1936)	1,378 (502, 1.2 ×10^4^)	1,221 (479, 5,467)
	Posterior hippo	567 (233, 2,358)	1,181 (452, 6,813)	985 (392, 3,904)
	Whole hippo	**460 (191, 1,566)**	**1,113 (446, 6,063)**	1,034 (414, 4,255)
	ERC	549 (202, 2,889)	2024 (570, 7.0 ×10^4^)	767 (342, 2,569)
	BA35	1,032 (345, 1.3 × 10^4^)	1927 (626, 2.8 × 10^4^)	**455 (220, 1,078)**
	BA36	2,802 (635, 8.4 × 10^5^)	8,347 (1,369, 3.5 × 10^6^)	1,579 (562, 1.0 × 10^4^)
	PHC	760 (254, 5,988)	3,485 (785, 5.0 × 10^5^)	1,369 (466, 8,725)
	Longitudinal change in other markers of neurodegeneration			
	Plasma NfL	2,302 (530, 1.8 × 10^5^)	6,136 (1,396, 1.2 × 10^6^)	46,848 (1,560, 9.6 × 10^6^)
	Longitudinal change in cognition			
	PACC	4,787 (598, 1.8 × 10^6^)	6,838 (975, 1.9 × 10^6^)	6,701 (1,305, 1.4 × 10^6^)
	ADAS‐Cog	11,523 (729, 2.9 × 10^6^)	50,585 (1,630, 6.8 × 10^6^)	62,853 (2,467, 1.2 × 10^7^)

*Note:* The best measure for each patient group was highlighted in bold font.

Abbreviations: AD, Alzheimer's disease; ADAS‐Cog, Alzheimer's Disease Assessment Scale‐Cognitive; BA35/36, Brodmann area 35/36; CSF, cerebrospinal fluid; EMCI, early mild cognitive impairment; ERC, entorhinal cortex; Hippo, hippocampus; NfL, neurofilament light chain; PACC, preclinical Alzheimer's cognitive composition; PHC, parahippocampal cortex.

### 3.4 Difference in MTL subregion atrophy in preclinical AD

**FIGURE 3:**
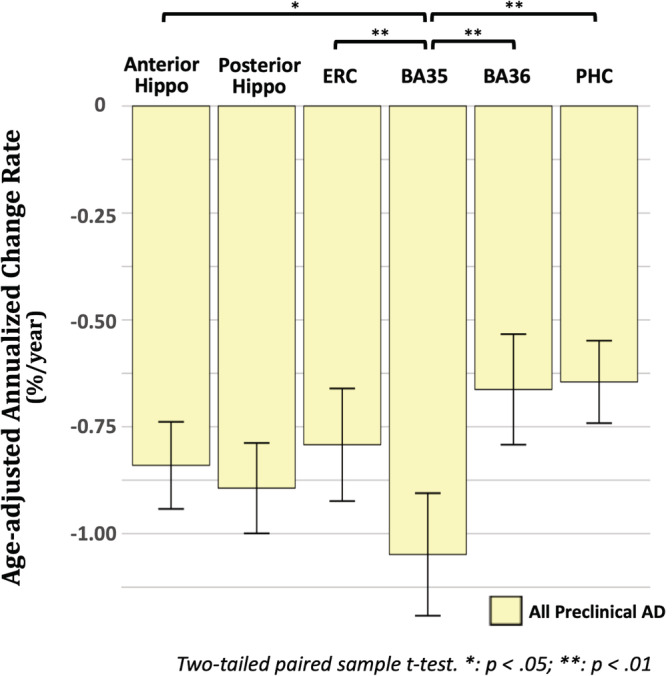
Comparisons of the longitudinal atrophy rate of different MTL subregions in preclinical AD. AD, Alzheimer's disease; Aβ, amyloid‐β; BA35/36, Brodmann area 35/36; ERC, entorhinal cortex; Hippo, hippocampus; PHC, parahippocampal cortex

From the longitudinal analysis, we observed that the atrophy rate of BA35 was faster than that of the other subregions in preclinical AD patients (Figure 3). We performed a post hoc exploratory analysis to investigate whether this effect was statistically significant. Two‐sided paired *t*‐tests showed that BA35 had significantly faster atrophy rate than the other MTL cortical subregions (ERC [*t* = 2.7, *p* = .008], BA36 [*t* = 3.3, *p* = .002] and PHC [*t* = 3.2, *p* = .002]) and anterior hippocampus (*t* = 2.2, *p* = .030). The differences between BA35 and posterior hippocampus did not reach significance.

## 4 DISCUSSION

In this study, using a highly tailored MRI processing pipeline that accounts for common confounds (Figure 1) in the quantification of longitudinal atrophy of MTL subregions, we were able to sensitively detect neurodegeneration in preclinical AD. Experimental results demonstrated that the rate of longitudinal change of hippocampus (both anterior and posterior), ERC and BA35 in preclinical AD are significantly faster than that of Aβ− controls. In addition, significant group differences were observed in the T+, but not T− subgroup, which supports the notion that accelerated atrophy in preclinical AD occurs only in the presence of concomitant tau pathology. Overall, our longitudinal measures of the MTL subregions were more sensitive to disease progression than longitudinal cognitive measures (PACC and ADAS‐Cog) and plasma NfL. Finally, we found that longitudinal measurements better discriminated preclinical AD from controls compared to cross‐sectional measures, confirming our hypothesis that the former is less susceptible to inter‐subject variability by non‐AD factors, such as developmental differences and the sum total of other modulators of structure throughout the lifespan.

### 4.1 Longitudinal atrophy measures and atrophy pattern are generally consistent with the literature

In this study, we observed a 0.63%/year hippocampal atrophy rate in cognitively normal controls regardless of Aβ status, which is similar to that of the 10 studies using automated processing methods summarized in a meta‐analysis of longitudinal atrophy in normal older adults (Fraser, Shaw, & Cherbuin, 2015), the 0.72%/year in (Holland, McEvoy, Desikan, & Dale, 2012), and the 0.59%/year reported by our prior study using longitudinal T2‐weighted MRI (Das et al., 2012). Notably, studies using manual segmentation methods reported higher atrophy rates (Fraser et al., 2015). In early prodromal AD (Aβ+ EMCI), we observed a 1.03%/year atrophy rate, which is lower than that of prior studies of MCI (Chincarini et al., 2016; Das et al., 2012; Holland, McEvoy, Desikan, & Dale, 2012; Iglesias et al., 2016; Jack et al., 2000; Kulason et al., 2019; Ledig et al., 2018; Leung et al., 2010; Morra et al., 2009; Schuff et al., 2009; Tward et al., 2017; Wolz et al., 2010) (range from 1.55%/year to 3.2%/year). This discrepancy is likely due to segmentation protocol and analysis methodology differences and/or the fact that we only included EMCI patients, who are expected to be at an earlier disease stage than most MCI cohorts in other studies.

While the hippocampus has long been the focus of biomarker research, ERC and BA35 are the first cortical sites of NFT pathology (Braak & Braak, 1995) and have been less frequently included in longitudinal analyses. In general, our longitudinal atrophy rates in ERC (0.43, 0.79, 1.18%/year) and BA35 (0.65, 1.05, 1.56%/year in Aβ− controls, preclinical AD, and Aβ+ EMCI respectively) are smaller than that in prior studies using automatic longitudinal quantification methods (ERC in Holland, McEvoy, Desikan, & Dale, 2012: 0.68, 0.89 and 2.46%/year in Aβ− controls, preclinical AD, and Aβ+ MCI; ERC in Miller et al., 2013: 1.0 and 2.7%/year in controls and preclinical AD; ERC and transentorhinal cortex in Kulason et al., 2019: 1.3 and 5.87%/year in controls and MCI; transentorhinal cortex in Tward et al., 2017: 2.35 and 6.42%/year in controls and MCI), probably due to, again, differences in segmentation protocol, definition of preclinical AD, the use of EMCI rather than a more typical MCI cohort, and the length of follow‐up time.

Being able to measure atrophy rates of granular MTL subregions allows for investigation into the spatial pattern of atrophy, which is a unique aspect of our processing pipeline. When comparing preclinical AD to Aβ− controls, we found significant differences in multiple MTL subregions, which is consistent with the results reported by prior studies (Miller et al., 2013; Pegueroles et al., 2017). Across the subregions, BA35 exhibited the greatest volume loss in absolute terms, followed by ERC and hippocampus. BA36 and PHC had the slowest atrophy rate among the MTL subregions in absolute terms. This result fits well with the pattern of spreading of NFT (Braak & Braak, 1995), that is, beginning at the transentorhinal region (approximates the BA35 in our segmentation protocol), spreading to ERC and hippocampus and then to the BA36. Thus, this pattern supports the notion that our longitudinal measurements are sensitive to tau‐mediated neurodegeneration, along with this effect found only in the T+ preclinical AD subgroup.

Of note, while BA35 demonstrated the largest atrophy rate of any region in the preclinical AD group (significant compared to ERC, BA36, PHC and anterior hippocampus while only in absolute terms compared to posterior hippocampus, Figure 3) and had a marginally larger absolute difference with Aβ− controls compared to that of the hippocampal measurements, the latter had stronger statistical significance (smaller *p*‐value). This is likely due to the larger variability in measurement of BA35 atrophy compared to that of the hippocampus, likely because BA35 is much harder to measure due to its smaller size and variability of its location (depending on the collateral sulcus pattern shown in Figure 1). This suggests that by improving the accuracy of BA35 measurements through advances in imaging and image analysis technology, we could derive more sensitive biomarkers of preclinical AD in the future. Nonetheless, BA35 was the only region in the cross‐sectional analysis to approach significance in differentiating the preclinical group from the controls and its longitudinal change demonstrated the strongest statistical significance in separating early prodromal AD patients from Aβ− controls.

In this study, we did not observe significant group differences in longitudinal change of cognitive measures, including the PACC, which has previously demonstrated sensitivity to the cognitive decline of preclinical AD (Donohue et al., 2017). Indeed, the current finding may appear inconsistent that reported by Donohue et al. (2017), in which they found a significant group effect using the PACC for discrimination of preclinical AD from Aβ− controls. This difference likely stems from our using only a 2‐year follow‐up period. Indeed, Donohue et al. (2017) did not find a significant group difference at this time period and group separation did not become more apparent until 4–5 years of longitudinal follow‐up.

While not providing the degree of group discrimination as the MRI measures in the longitudinal analysis and exhibiting greater variability, plasma NfL findings were qualitatively quite similar with the exception of the early MCI group. In fact, the cross‐sectional analysis revealed a highly similar pattern of NfL to BA35. These findings continue to support prior promising findings of the potential value of this relatively noninvasive and high accessible blood‐based biomarker (de Wolf et al., 2020; Mattsson, Cullen, Andreasson, Zetterberg, & Blennow, 2019).

### 4.2 Accelerated atrophy in preclinical AD is associated with concomitant tau pathology

In this study, we only observed significant increases in atrophy rates in T+ preclinical AD patients, not T− ones, suggesting that MTL neurodegeneration only occurs in subjects with evidence of concomitant tau pathology. In fact, the magnitude of most of the MTL longitudinal measures in T+ preclinical AD patients is comparable to that of the early prodromal AD (except for BA35 which is larger in the early prodromal AD group). A similar finding was reported in prior cross‐sectional (Fortea et al., 2014) or longitudinal (Desikan et al., 2011; Holland, McEvoy, Desikan, & Dale, 2012; Pegueroles et al., 2017) studies, in which structural abnormality were only observed in subjects with evidence of both amyloid and tau pathologies. Our finding further supports the notion that, compared to amyloid pathology, tau pathology is more directly linked to neurodegeneration. Additionally, it also demonstrates that our longitudinal measurements are sensitive to tau‐mediated neurodegeneration.

### 4.3 Sample size estimation in MCI and preclinical AD

In this study, we computed sample size relative to controls (as in Cash et al., 2015; Das et al., 2012; Holland, McEvoy, & Dale, 2012; Leung et al., 2010) rather than to zero atrophy rate (as in Chincarini et al., 2016; Wolz et al., 2010), because the differential effect between the treatment and placebo groups is of greater interest in clinical trials. Our results shown in Table 4 suggest a considerable reduction in the number of subjects that would need to be enrolled relative to cognitive measures and plasma NfL in all patient groups.

A couple prior studies have reported sample size estimation in MCI cohort. Holland et al. reported 169 and 294 subjects (ERC, 2‐sided, ADNI‐1, 3‐year follow‐up) are needed to detect a 25%/year reduction in longitudinal change in MCI regardless of amyloid status and Aβ+ MCI patients, respectively (Holland, McEvoy, Desikan, & Dale, 2012). Other studies reported 286 (Holland, McEvoy, & Dale, 2012) (ERC, 2‐sided, ADNI‐1, 3‐year follow‐up), 545 (Leung et al., 2010) (hippocampus, 2‐sided, ADNI‐1, 1‐year follow‐up) and 269 (Das et al., 2012) (hippocampus, 1‐sided, UPenn dataset, within 3 years) subjects needed to detect a 25%/year reduction in longitudinal change in MCI regardless of Aβ status. The MCI cohort in these prior studies would mostly be considered late MCI using the ADNI‐GO/−2 definition rather than EMCI used in the current study. To better compare our processing pipeline with prior work, we additionally computed sample size for the Aβ+ late MCI patients (*n* = 77) and found that 91 (25%/year, one‐sided) and 116 (25%/year, two‐sided) subjects are needed using ERC atrophy rate. Nonetheless, comparison with prior studies is limited by differences in years of follow‐up and Aβ status.

Holland, McEvoy, Desikan, and Dale (2012) also performed sample size estimation for preclinical AD, where they reported *n* = 1,763 and *n* = 2,672 subjects (two‐sided) needed to detect a 25% reduction in longitudinal hippocampus and ERC change rates respectively. On the other hand, using the proposed longitudinal measurements, we achieved more than 3‐fold reduction in sample sizes [*n* = 586 and *n* = 700 (two‐sided, see Section S1.4 and Table S1) using hippocampus and ERC respectively], which may indicate the potential utility of our granular longitudinal measurements in future clinical trials.

### 4.4 Limitations and future work

The current study has several limitations. First, no hippocampal subfield measurements were extracted and compared because we cannot obtain reliable hippocampal subfields segmentation from T1w MRI due to limited tissue contrast of internal hippocampal structures (de Flores, La Joie, & Chételat, 2015; Wisse, Geert Jan Biessels, 2014). It will be interesting to investigate the pattern of longitudinal atrophy at the subfield level in future work using the growing dataset of specialized high resolution T2‐weighted MRI currently being acquired in ADNI‐3 and other studies. Second, CSF p‐tau measures may not does not specifically reflect regional tau burden in the MTL and thus the relationship between MTL tau deposition and longitudinal atrophy cannot be investigated here. Assessment of the relationship of regional MTL tau burden extracted from tau PET and MTL subregional atrophy measures, similar to Das et al. (2018, 2019), Xie et al. (2018), but in the preclinical AD, should be done in the future work when sufficient tau PET data is available. Third, in addition to the morphometric analysis used in this study, other measurements, such as shape and appearance‐based measures (e.g., texture), could be explored in future work. Fourth, although symmetric registration is crucial to reduce the bias in atrophy rate estimation, it may introduce some additional variability pointed out by Tward, Mitra, and Miller (2019).

## CONFLICT OF INTERESTS

Dr. Wolk received grants from Eli Lilly/Avid Radiopharmaceuticals, personal fees from Eli Lilly, grants and personal fees from Merck, grants from Biogen, personal fees from Janssen, and personal fees from GE Healthcare. Dr. Xie received personal consulting fees from Galileo CDS, Inc. Dr. Das received personal fees from Rancho Biosciences.

## Supporting information


**Appendix S1** Supporting Information.

## Data Availability

The data that support the findings of this study are available in Alzheimer's Disease Neuroimage Initiative (ADNI) at adni.loni.usc.edu.

## Endnotes

^1^ Available publicly in the processed data on the ADNI website: adni.bitbucket.io/reference/docs/UCBERKELEYAV45/ADNI_AV45_Methods_JagustLab_06.25.15.pdf

^2^ Automatic Segmentation of Hippocampal Subfields‐T1 (ASHS‐T1) is an open‐source software that performs automatic segmentation of medial temporal lobe subregions from T1‐weighted MRI. Detail description is available at https://sites.google.com/view/ashs-dox/home
