# Supplementary material for: Longitudinal atrophy in early Braak regions in preclinical Alzheimer's disease
Source: Hum Brain Mapp. 2020 Aug 26;41(16):4704–17. doi: 10.1002/hbm.25151 (PMC7555086; doi:10.1002/hbm.25151)
Supplement: Supplementary file 1 — Appendix S1 Supporting Information. [file HBM-41-4704-s001.docx]

**Supplementary Material**


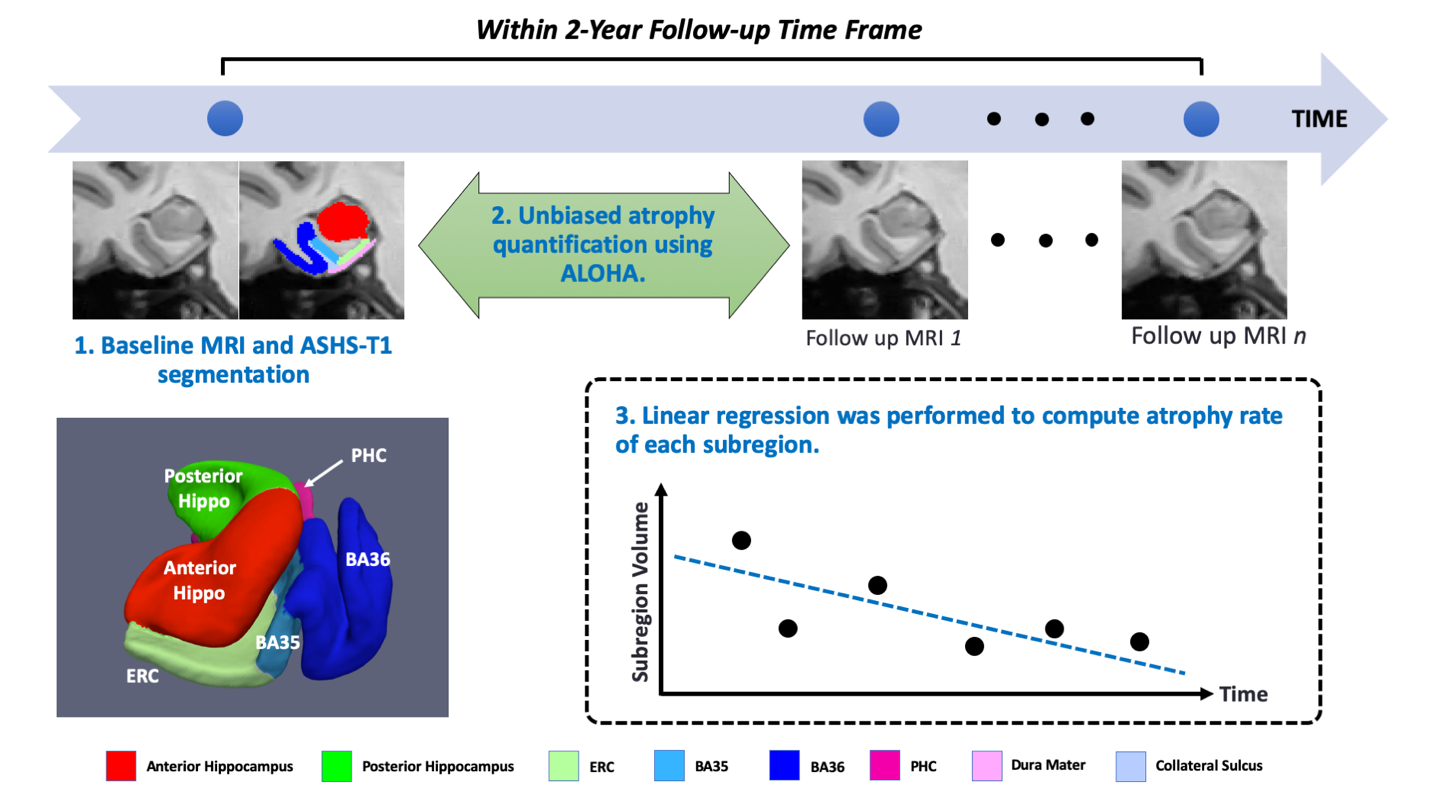


**Supplementary Figure S1.** The three steps of the longitudinal atrophy rate estimation pipeline: 1. Segment the medial temporal lobe (MTL) subregions using ASHS-T1. 2. Estimate volume of the follow-up timepoints using ALOHA, an unbiased registration-based longitudinal pipeline. 3. Derive the volume atrophy rate of each subregion by performing linear regression to the longitudinal volume measurements. The model can be fit using only 2 timepoints. ERC = entorhinal cortex; BA35/36 = Brodmann area 35/36; Hippo = hippocampus; PHC = parahippocampal cortex; ASHS-T1 = automatic segmentation of hippocampal subfield-T1 software; ALOHA = automatic longitudinal hippocampal atrophy.

**S1. Details of the method**

**S1.1. The ADNI study**

Data used in this study were obtained from the Alzheimer’s Disease Neuroimaging Initiative (ADNI, adni.loni.usc.edu). The ADNI was launched in 2003 as a public-private partnership, led by Principal Investigator Michael W. Weiner, MD. The primary goal of ADNI has been to test whether serial magnetic resonance imaging (MRI), positron emission tomography (PET), other biological markers, and clinical and neuropsychological assessment can be combined to measure the progression of mild cognitive impairment and early Alzheimer’s disease. For up-to-date information, see www.adni-info.org.

**S1.2 Neuroimaging data acquisition and processing**

**S1.2.1 Imaging data acquisition**

The MRI scans were acquired from different scanners at multiple sites. Up-to-date information about MRI imaging protocols can be found at adni.loni.usc.edu/methods/mri-tool/mri-analysis. For Florbetapir PET, images were acquired for 20 minutes (4 frames of 5 minutes duration) after a 50-minute uptake phase following injection of 10 mCi of tracer. Further detail on the PET acquisition is available at adni.loni.usc.edu/methods/pet-analysis-method/pet-analysis.

**S1.2.2 Discussion on using longitudinal volume vs. cross-sectional thickness for MTL cortex subregions**

Thickness is used for cortical subregions (ERC, BA35, BA36 and PHC) because it is thought to be a less noisy measure than volume in the cross-sectional setting, as it is less sensitive to segmentation errors of subregion boundaries. This is less of an issue for longitudinal change (where we use volume rather than thickness) because it is quantified using pairs of MRI scans of the same subject with an unbiased deformable-registration-based pipeline, which should be minimally affected by uncertainty of subregion boundaries. Therefore, we used different measurements (volume and thickness) in different analysis (longitudinal and cross-sectional) for MTL cortical subregions.

**S1.3 Quality control**

Originally, 339 participants were included in this study. In order to ensure quality of the cross-sectional and longitudinal measurements, we performed comprehensive quality control to identify and exclude cases that were not processed successfully by the image processing pipeline. The quality control includes three levels: the MRI scans, the ASHS-T1 automatic segmentation, and the longitudinal estimation. In the first step, all the baseline MRI scans were checked visually and those with severe motion artifact or blurring were excluded. Two subjects (1 Aβ- control and 1 early prodromal AD patient) were excluded in this step.

To control for the quality of automatic segmentation, we visually check the ASHS-T1 outputs for all subjects. There were small errors in a subset of individuals. We observed under-segmentation of the lateral border of the hippocampus in 17 out of 337 subjects (7 Aβ- control, 2 preclinical AD, 8 early prodromal AD) and over-segmentation of MTL cortex in 1 out of 337 subjects (Aβ- control). No subject had both kind of errors. These participants were included for analyses using only the accurately segmented region (i.e. if the hippocampal segmentation was poor, it was excluded from analyses using this region but all other regions that passed QC were kept in the analyses).

In addition, the quality of longitudinal estimation was assessed. Scans of follow-up timepoints that were poorly registered to the baseline were excluded from the atrophy rate estimation. However, due to the very large number of registration pairs, it was not feasible to visually check all. Instead, we checked a subset of registrations selected using the following method: (1) we computed the normalized cross-correlation (NCC) of the registered scans, which is a good indicator of registration quality, and then selected pairs of registrations that yielded NCC that are 1.5 standard deviation below the mean. (2) We also randomly selected 5% of the remaining pairs to confirm that they had high registration quality.

After quality control, data from 337 participants were analyzed and reported in the main text.

**S1.4 Sample size calculation**

As in prior longitudinal studies, we estimate the sample size required to detect both $C=50\%/year$ and $C=25\%/year$ reduction in the atrophy rate of each patient group relative to the atrophy rate of Aβ- controls (significance level α = 0.05, power 1-β = 0.8) using Equation (1), where $\bar{A}_{PAT}$ and $\bar{A}_{CTL}$ are mean atrophy rates in patients and Aβ- controls respectively, $z_{t}$ is the $t^{th}$ quantile of the standard normal distribution, and $S_{PAT}$ is the standard deviation of the patient atrophy rate [Bhalerao and Kadam, 2010]. The 95% confidence interval of each sample size estimate is computed using the bootstrap method [Efron, 1979]. Similarly, we estimated sample size using one-sided test in our primary analysis (reported in Table 4) due to our strong hypothesis on the direction of the effect. However, since prior studies commonly performed two-sided tests, we additionally computed two-sided sample sizes for better comparisons (reported in Supplementary Table S1).

|  | $N=2\left[ \frac{(z_{\alpha}+z_{1-\beta})S_{PAT}}{C*(\bar{A}_{PAT}-\bar{A}_{CTL})} \right]^{2}$ | (1) |
| --- | --- | --- |

**Supplementary Table S1.** Sample size (95% confidence interval in parenthesis) required to detect both 50%/year and 25%/year reduction in change rate of each patient group compared to that of Aβ- controls (power 1-β = 0.8, two-sided significance level α = 0.05). The best measure for each patient group was highlighted in fold font.

| **% Change** | **Measurement** | **Preclinical AD** | | **Early Prodromal AD**  **(A+ EMCI)** |
| --- | --- | --- | --- | --- |
|  |  | **CSF p-tau Positive**  **(A+T+ Controls)** | **Whole Group**  **(A+ Controls)** |  |
| **50%** | **Longitudinal atrophy rate** | | | |
|  | Anterior Hippo | 160 (66, 589) | 435 (161, 4177) | 389 (150, 1857) |
|  | Posterior Hippo | 181 (73, 751) | 504 (195, 2968) | 420 (169, 1705) |
|  | Whole Hippo | **147 (62, 507)** | **355 (139, 1850)** | 330 (133, 1352) |
|  | ERC | 175 (63, 937) | 645 (186, $2.0\times{10}^{4}$) | 244 (111, 816) |
|  | BA35 | 329 (107, 4098) | 614 (201, 8904) | **145 (71, 343)** |
|  | BA36 | 632 (165, $4.4\times{10}^{4}$) | 1549 (358, $2.8\times{10}^{5}$) | 450 (168, 2375) |
|  | PHC | 192 (68, 1076) | 708 (198, $2.3\times{10}^{4}$) | 392 (137, 2103) |
|  | **Longitudinal change in other markers of neurodegeneration** | | | |
|  | Plasma NfL | 734 (175, $5.7\times{10}^{4}$) | 1955 (451, $4.1\times{10}^{5}$) | 14929 (516, $3.4\times{10}^{6}$) |
|  | **Longitudinal change in cognition** | | | |
|  | PACC | 1526 (198, $4.5\times{10}^{5}$) | 2179 (331, $5.1\times{10}^{5}$) | 2136 (425, $4.0\times{10}^{5}$) |
|  | ADAS-Cog | 3652 (228, $1.1\times{10}^{6}$) | 16120 (538, $3.1\times{10}^{6}$) | 20030 (758, $3.8\times{10}^{6}$) |
| **25%** | **Longitudinal atrophy rate** | | | |
|  | Anterior Hippo | 638 (266, 2394) | 1757 (632, $1.7\times{10}^{4}$) | 1556 (618, 7263) |
|  | Posterior Hippo | 723 (290, 2994) | 1506 (567, 8858) | 1256 (493, 4916) |
|  | Whole Hippo | **586 (247, 1999)** | **1419 (565, 7516)** | 1319 (531, 5056) |
|  | ERC | 700 (253, 3678) | 2580 (725, $1.0\times{10}^{5}$) | 978 (429, 3201) |
|  | BA35 | 1316 (440, $1.7\times{10}^{4}$) | 2457 (813, $3.5\times{10}^{4}$) | **580 (283, 1389)** |
|  | BA36 | 2530 (673, $1.5\times{10}^{5})$ | 6196 (1418, $1.1\times{10}^{6}$) | 1799 (655, $1.0\times{10}^{4})$ |
|  | PHC | 766 (277, 4000) | 2831 (805, $1.1\times{10}^{5}$) | 1567 (538, 9025) |
|  | **Longitudinal change in other markers of neurodegeneration** | | | |
|  | Plasma NfL | 2934 (689, $2.8\times{10}^{5}$) | 7821 (1789, $1.7\times{10}^{6}$) | 59717 (2025, $1.1\times{10}^{7}$) |
|  | **Longitudinal change in cognition** | | | |
|  | PACC | 6102 (774, $1.8\times{10}^{6}$) | 8716 (1259, $3.1\times{10}^{6})$ | 8542 (1734, $1.8\times{10}^{6}$) |
|  | ADAS-Cog | 14609 (916, 4$.3\times{10}^{6}$) | 64481 (2112, $1.6\times{10}^{7}$) | 80120 (3077, $1.0\times{10}^{7}$) |

Note: AD = Alzheimer’s disease; Hippo = hippocampus; ERC = entorhinal cortex; BA35/36 = Brodmann area 35/36; PHC = parahippocampal cortex; PACC = preclinical Alzheimer’s cognitive composition; ADAS-Cog = Alzheimer’s disease assessment scale – cognitive; CSF = cerebrospinal fluid; NfL = neurofilament light chain.

**References**

Bhalerao S, Kadam P (2010): Sample size calculation. Int J Ayurveda Res 1:55. https://www.ncbi.nlm.nih.gov/pmc/articles/PMC2876926/.

Efron B (1979): Bootstrap Methods: Another Look at the Jackknife. Ann Stat 7:1–26.
